# Protective versus Risk Factors for 18-month Outcomes in a multi-decade sample of preterm infants age 23–30 weeks

**DOI:** 10.18103/mra.v13i10.7027

**Published:** 2025-10-28

**Authors:** Ted S. Rosenkrantz, Ruth M. McLeod, R. Holly Fitch

**Affiliations:** 1. Dept of Pediatrics, University of Connecticut School of Medicine and Connecticut Childrens Medical Center; 2. Dept. of Psychology, Kutztown University, Kutztown, PA, 19530; 3. Dept. of Psychological Sciences/Behavioral Neuroscience, University of Connecticut, Storrs, CT, 06268.

**Keywords:** Prematurity, birthweight, magnesium sulfate, methylxanthine, cognitive, motor

## Abstract

This paper summarizes risk and protective factors modulating cognitive, language and motor outcomes in a cohort of preterm infants from Connecticut Children’s Medical Center and University of Connecticut Health Center. Infants were born at 23–30 weeks gestational age and admitted to the neonatal unit (1991 – 2017). Extracted and de-identified patient data included sex, gestational age (GA), birthweight, maternal health conditions (pre-eclampsia, diabetes, etc.), presence of necrotizing enterocolitis, intra-ventricular hemorrhage and grade, maternal magnesium sulfate (MGS) treatment, and administration of the methylxanthine (MX) adenosine antagonists (caffeine or theophylline) and timing (< 48 hrs from birth (early) or > 48 hrs (late)). Outcome measures were obtained at 18-month follow-up evaluations (Bayley Scale and/or Cognitive Adaptive Test/Clinical Linguistic and Auditory Milestone Scale). Scores on different components of the tests were z-scored and averaged into 3 categories for each infant as Language, Cognitive, and Motor indices.

Significant risk factors for *poor* outcomes were found to include: (1) being male, (2) extremely low birthweight (a better predictor of poor outcome than low GA), (3) positive inflammatory perinatal profile; and (4) MGS exposure in males, particularly when followed by early MX treatment. Factors leading to significantly *better* outcomes included: (1) MX exposure within 48 hours of birth, *particularly* in infants with inflammation (but excluding males with prior MGS exposure); and (2) perinatal MGS exposure, *particularly* in lower birthweight females (but excluding early MX-treated males). This novel evidence of sub-group specific therapeutic benefits and harms emphasizes a serious need for additional research on individualized therapeutic interventions for at-risk preterm infants and reveals a novel deleterious interaction between magnesium sulfate exposure and subsequent treatment with methyxanthines (e.g., caffeine) within 48 hours of birth for preterm boys.

## Introduction

Infants born prematurely (gestational age (GA) under 38 weeks) are at increased risk for health complications including neurologic, cardio-vascular and pulmonary function. These risks become more pronounced with decreasing GA/birthweight and can combine to restrict oxygen and energy supply to the developing brain^1, 2, 3^. Postnatal events can include chronic hypoxia resulting from respiratory insufficiency, as well as hypoxic-ischemic neural events associated with blood pressure instability leading to hemorrhagic and/or ischemic neural events (e.g., intra-ventricular/periventricular hemorrhage or IVH/PVH;^4, 5^). These conditions can be further complicated by metabolic and/or thermoregulatory insufficiency, as well as inflammatory factors present due to maternal-fetal conditions such as pre-eclampsia, maternal diabetes, chorioamnionitis and/or postnatal necrotizing enterocolitis (NEC;^6, 7, 8^). Hypoxic-ischemic and other inflammatory events are in turn associated with increased mortality and morbidity, with long-term effects including heightened incidence of cerebral palsy (CP), intellectual disability, attention deficits, and academic disabilities^9–12^. From a clinical perspective, an improved understanding of the relationships between:^1^ specific preterm risk factors and poor outcomes (particularly using individualized cognitive, language and motor outcomes in favor of coarser measures such as morbidity/mortality); as well as^2^ mitigating variables and improved outcomes; could significantly aid in the optimization of neonatal care for at-risk premature infants.

Prior work has identified a number of specific risk factors shown to worsen outcomes for high-risk infants, such as the incidence of IVH-PVH, with increasing severity of injury directly related to increased mortality and morbidity. Likewise, GA is a well-established risk that increases the risk of poor outcomes with decreasing GA. In addition to these well-established risks, other more subtle risk factors have been identified which could be of medical import and should be critically considered as part of maternal-fetal and neonatal care. These include risks associated with being male (or alternately, protection associated with being female^13^), as well as risks associated with increased circulatory presence of inflammatory factors due to injury, infection or other conditions affecting the pregnant mother and/or newborn^14–17^.

On the other hand, potential therapeutic interventions are also of substantial interest. Unfortunately, whereas an approved therapeutic treatment exists for full-term infants experiencing perinatal hypoxic-ischemic brain injury (a 3-day regimen of head or whole-body hypothermia or “cooling”), this therapy has not been shown to benefit preterm infants and is not approved for the preterm population^18^. No other therapeutic interventions have been FDA approved to address neurologic risks and long-term neurobehavioral impacts in preterm infants^19, 20^. Nonetheless, retrospective clinical and pre-clinical animal studies have identified several pharmacologic agents that may provide neuroprotection with long-term benefits. These include early exposure to methylxanthines (caffeine/theophylline), magnesium sulfate, erythropoietin, xenon, argon, melatonin and others^21, 22^. Caffeine and magnesium sulfate are of particular interest since they are among only a handful of drugs already approved for maternal-fetal and neonatal use^23, 24, 25^.

The current study assessed a cohort of 784 preterm infants (23–30 weeks) whose clinical data was ascertained retrospectively and assessed at Connecticut Children’s Medical Center (CCMC) and the University of Connecticut Health Center (UConn Health). Independent variables included sex, gestational age (GA), birthweight, maternal health conditions (pre-eclampsia, diabetes, etc.), presence of necrotizing enterocolitis (NEC), intra-ventricular hemorrhage (IVH and grade), maternal magnesium sulfate (MGS) treatment, and administration of the adenosine antagonists caffeine or theophylline (methylxanthines, MX), as well as whether these were administered < 48 hrs of birth (“early”) or > 48 hrs (“late”). Dependent variables comprised outcome measures obtained at 18-month follow-up evaluations. Findings confirm some previously reported risks and benefits as measured by outcome, but also reveal important new evidence of sub-group specific benefits and harms.

## Methods

The full dataset used in the current analyses was described in detail in previous publications^26, 27^. All methods were approved by the applicable Institutional Review Board (IRB). Connecticut Children’s Medical Center IRB #19-112 and The University of Connecticut Health Center IRB 19X-211-1.

### SUBJECTS & DATA COLLECTION

Subjects were infants born at UCHC and cared for at the University of Connecticut Health Center (UCHC) / Connecticut Children’s Medical Center (CCMC) NICU between Jan 1991 and Dec 2017. From the total infants born in this period at 23 – 30 weeks, we only included infants who also had an ~18-month neurodevelopmental follow-up (n = 784) which was the majority of infants (>80%). Data were collected by trained NICU nurses and stored in a computerized database, the *Neonatal Information System 3* (Medical Data Systems, Phil, PA, USA). Select variables were collected from this original clinical data including: GA, preeclampsia, chorioamnionitis, perinatal magnesium sulfate, mode of delivery, birth year, sex, birth weight, Apgar score, cord pH, Snap score, IVH and grade, length of stay, type of treatment, and length of treatment. These measures were selected for evaluation based on known or suspected impact on long-term outcomes. Clinical definitions and dosing parameters for these measures and treatments did not change substantially over the study period, but we further divided subjects into three-decade groups to control for possible changes in medical care (1991–2000, 2001–2010, and 2011–2017). As this was a retrospective study, consent was not obtained. Subjects were deidentified at the time of data collection, and no identifiable information was collected, with subjects assigned a new subject number. Behavioral and cognitive outcomes came from follow-up visits at 18 months of age (corrected to GA at birth). Data from the visits were collected and stored in the High-Risk Follow-Up section of the NIS database and the CCMC NICU Neurodevelopmental Follow-up Clinic database. Outcomes were measured using the Bayley II and III or Cognitive Adaptive Test/Clinical Linguistic and Auditory Milestone Scale (CAT/CLAMS) assessments. Only infants who had at least cognitive and language outcome measures were included in this study (81% retention from the base sample, final n = 784). Multiple editions of the Bayley Scales were available during the study period (Bayley II–III), with infants receiving either the Bayley III (25%), the Bayley II (14%), the CAT/CLAMS (60%), or both the Bayley II and CAT/CLAMS (1%). We chose to Z-score relevant sub-scale measures as available for each subject, and average these into domain scores in language, motor and cognition (the number of infants with social indices was insufficient for analysis). For the Bayley, sub-scales included Gross Motor, Fine Motor, Problem Solving, Expressive Language, Receptive Language, Language Articulation, Self-Help, and Relationships with Others; for the CAT/CLAMS (the Cognitive Adaptive Test (CAT) and the Clinical Linguistic Auditory Milestone Scale (CLAMS)), sub-scales included Cognitive and Language scores. Extraction of sub-scores was necessary because not all subjects received all tests (due to infant drop-out, non-participation during the exam, etc.), resulting in partial profiles. This standardization also helped to control for comparison across test versions. Raw scores from all sub-scales were converted to z-scores for each subject (performed independently for each assessment sub-test within each of the three decades to control for evolving medical practices). These sub-scale Z-scores were averaged into four categories or domains —Social (comprised of the Self-help and Relationship with others Z-scores), Cognitive (comprised of Problem Solving, and the CAT Z-scores), Language (comprised of Expressive Language, Receptive Language, Language Articulation, and the CLAMS Z-Scores), and Motor (comprised of Gross Motor and Fine Motor Z-scores). Social scores were later dropped from analysis due to very low n.

### STATISTICAL ANALYSIS

Final values were entered into SPSS 28 (IBM) for analysis. ANCOVA’s (using Decade as a covariate for all analyses) were used to assess the effects of independent variables including: Sex (2 levels: male, female), GA (2 levels: 1=23–27 weeks; 2=28–30 weeks based on median split), Birthweight (2 levels; 1=<800 grams, 2=>799 grams based on median split), Inflammatory Category (2 levels; 1 = any of pre-eclampsia, diabetes, chorioamnionitis, necrotizing enterocolitis (NEC), or intra-ventricular hemorrhage (IVH), 0 = none), maternal magnesium sulfate treatment (MGS; y=1/n=0), and administration of the methyxanthine adenosine antagonists caffeine or theophylline (MX), as well as whether these were administered < 48 hrs (“early”) or > 48 hrs (“late,”). Specifically, we used the variables MX with 3 levels (none, early, late), as well as E/L only (early=0/late=1). Dependent variable outcome measures derived from 18-month follow-ups using averaged z-scores for each infant in the domains of: Language, Cognition, and Motor. (Social indices were also obtained for a small subset of infants, but n’s were insufficient for meaningful analysis). Additional correlations were run between continuous independent variables (GA, birthweight, and Inflammatory Index (0–5; chorio (y/n), diabetes (y/n), pre-eclampsia (y/n), IVH (y/n), or NEC (y/n), with each “yes” counting as 1), and cognitive, motor and language outcome scores.

We initially performed univariate ANCOVAs (SPSS) to evaluate the main effects of various categorical variables on the three outcome scores. We used individual ANCOVAs for Cognitive, Language and Motor outcome scores due to the differing n’s (that is, because multivariate analyses reduce the subject pool to infants with values for all outcome measures). In all analyses, Decade was used as a covariate to control for possible changes in medical practices. The effects of GA, birthweight, Inflammation, MGS, and Early/Late MX were also analyzed as a function of Sex, and further analyses on specifically defined sub-groups were performed to evaluate more complex multivariate interactions. Near-significant/marginal effects (*p*≤*.1*) are reported in italics.

## Results

**Figure 1.**
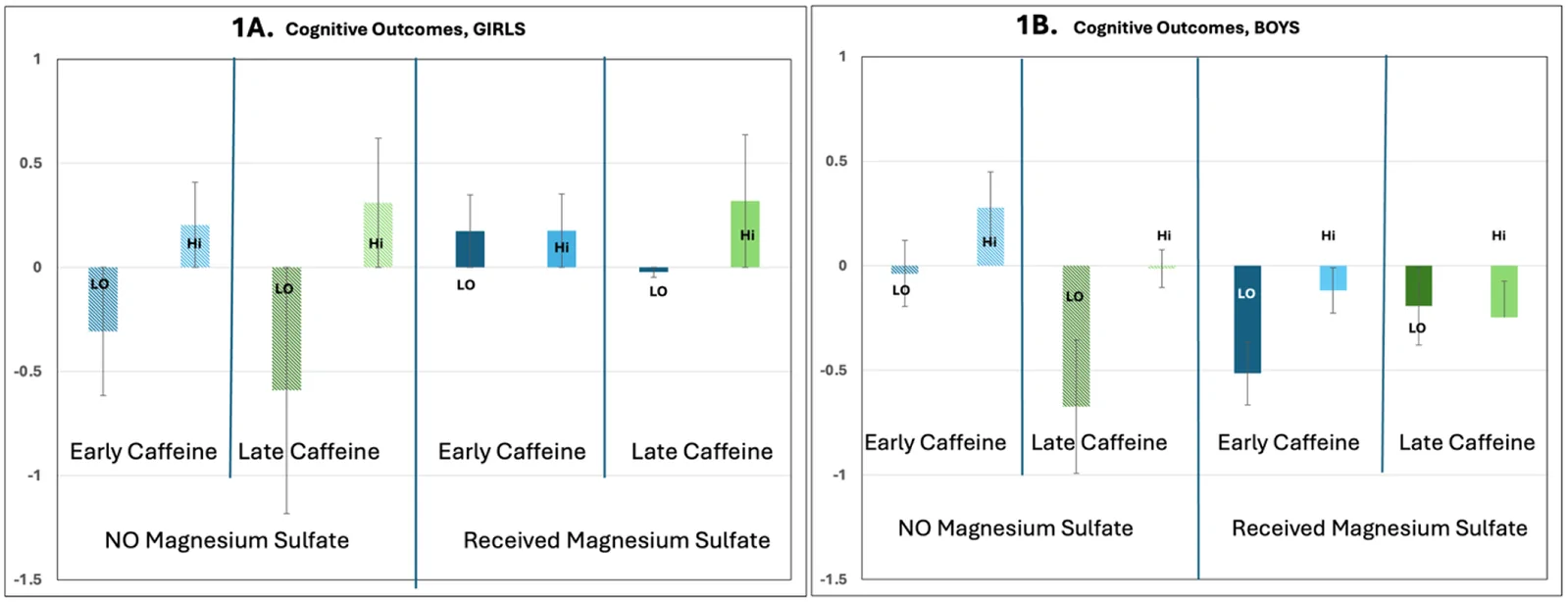
Mean cognitive outcomes as a function of sex, birthweight and drug treatment. Cognitive outcome scores for girls and boys as a function of birthweight (HI=>799 grams, LO=<800 grams based on median split); overall, girls performed better than boys. **1A.** For girls, HI birthweight led to better outcomes. Among those with LO birthweight, MGS+ was beneficial. **1B**. For boys, those with early caffeine and no MGS did best, while MGS+ led to significant harm for boys with early MX. Similar patterns are seen for Language and Motor outcomes (data not shown).

The following are results for effects of specific independent variables on 18-month outcomes (dependent variables). For **Sex** (2 levels), as previously reported^26^, a main effect was seen for Cognitive outcomes (p=.003, F=8.9 (1,773)), with girls outperforming boys (Language and Motor n.s.; see Figure 1).

For **Gestational Age** (GA, 2 levels, median split at 27/28 weeks), significant main effects were seen on Cognitive outcomes (p=.05, F=3.8(1,773)), marginal effects on Language (*p=.1*, F=2.7(1,777)), and significant effects on Motor (p=.007, F=7.37(1,283)). In all cases a lower GA predicted worse scores. A GA x Sex interaction was significant for Motor outcomes (p=.014, F=6.3 (1,281)), reflecting stronger overall GA effects in girls (GA effects in girls: Cognitive p=.014, Language p=.05, Motor p=.001; GA effects in boys, ns).

For **Birthweight** (BW, 2 levels, median split 800 g), highly significant overall main effects were seen on Cognitive outcomes (p<.001, F=19.4 (1,773), as well as Language (p=.002, F=9.36 (1,777)) and Motor (p<.001, F=11.76 (1, 283)) scores, with lower birthweight predicting worse scores. A marginal BW X Sex interaction was seen for Motor outcomes (*p=.1*, F=2.5 (1,281)), with stronger birthweight effects on motor outcomes in girls (Motor p=.001) compared to boys (Motor, ns). Pearson bivariate cross-correlations also revealed that birthweight strongly predicted outcomes in girls (BW/cognitive, R=.16, p=.003 (n=343); BW/language, R=.12, p=.016 (n=347); BW/motor, R=.3, p=.001 (n=139)), whereas birthweight only correlated significantly with cognitive outcomes in boys (R=.17, p=.001 (n=392)). BW/outcome correlations were largely unaffected by MX and/or MGS treatment (see Tables 1, 2, 3), suggesting the deleterious effects of low birthweight were not broadly mitigated by treatment. The exception to this was for low-birthweight girls treated with MGS, where the impacts of birthweight were weakened (Figure 1). This is consistent with particularly robust beneficial MGS effects in low birthweight girls.

For **Inflammatory Category** (2 levels, +/−), we saw no overall main effects, nor were effects seen separately in girls or boys. However, when we assessed *only* infants who received late MX *and* no MGS (both sexes), we found that inflammation exerted a significant negative effect on Language outcomes (p=.015, F=6.1(1,118)) and a marginally negative effect on Motor outcomes (*p=.1*, F=2.6 (1,53)). Subsequent analyses showed a strong negative correlation between Inflammatory Index (0–5) and outcomes in this low-treatment subset (Inflammatory Index/Language, R= −.26, p=.004 (121); Inflammatory Index/Motor, R= −.34, p=.01 (56)). Negative correlations between inflammatory status and outcomes were also quite robust among males, females, and combined-sex groups in the absence of *either* early MX *or* MGS (see Tables 1, 2, 3). Conversely, inflammatory status provided no correlations with outcomes among groups that received *either* early MX *or* MGS treatment (Tables 1, 2, 3).

For **Early/late methylxanthines**, as previously reported^27^, we saw no main effects of overall MX treatment (3 levels; none, early, late), but we saw overall benefits when specifically comparing early versus late methylxanthine treatment (E/L). Effects were seen for Cognitive outcomes (*p=.08*, one-tail, F=1.9 (1,685); Language and Motor, n.s.), with earlier treatment leading to better outcomes (Figure 1). As previously reported, these early MX benefits were much stronger among infants with an inflammatory history (chorioamnionitis, pre-eclampsia, maternal diabetes, IVH-PVH, NEC), as shown by a significant E/L X Inflammation Category interaction (Cognitive p=.05, F=3.9 (1,683); Language (ns); Motor, p=.026, F=5, (1,277)). When assessed separately, infants with inflammatory history showed early MX benefits (Cognitive, p=.022, F=5.3(1,358), Language and Motor, n.s.), none of which reached significance among infants with *no* inflammatory history. Benefits of early MX were also seen among infants with a low GA (<27 weeks) and an inflammatory history (Cognitive, p=.01, F=6.8 (1,194), Language and motor (n.s.)).

For **Magnesium Sulfate** (MGS, 2 levels), we also saw no overall main effects. However, as previously reported^26^, we did see highly significant Sex X MGS interactions (Cognitive, p=.002, F=9.7(1,771); Language, p=.01, F=6.6 (1,775); Motor, *p=.057*, F=3.7 (1,281, Figure 1)).

For girls only, we saw *beneficial* MGS effects (Cognitive, p=.022, F=5.3 (1,381), Language, n.s., Motor, *p=.056*, F=3.7 (1,136)). Interestingly, in girls we also found a highly significant interaction between MGS Treatment X Birthweight (Cognitive, p=.01, F=6.6 (1,379), Language, p=.031, F=4.7(1,382), Motor *p=.08*, F=3.13 (1,134)). Among low birthweight girls, we found robust benefits of MGS on Cognitive (p=.003, F=8.9 (1,154)), Language (p=.03, F=4.84 (1,154)) and Motor outcomes (p=.016, F=6.2(1,62)), whereas no significant benefits were seen for higher birthweight girls (though MGS-treated scores still trended better; Figure 1). Interestingly positive MGS effects in girls did not appear to relate to inflammatory status, as positive effects were seen both with and without a prenatal inflammatory history (among Inflam- girls, MGS effects on Cognitive, *p=.1*, F=2.5, (1,241), with MGS+ girls doing better; among Inflam+ girls, MGS effects on Cognitive, p=.042, F=4.2 (1,137) and Motor, p=.045, F=4.2 (1,53), again with MGS+ girls doing better).

Conversely, for boys we found significantly *deleterious* effects on Cognitive outcomes (p=.036, F=4.4 (1,389)) and Language outcomes (p=.016; F=5.8 (1,390); motor n.s.). Interestingly, these detrimental effects in boys differed depending on the timing of methylxanthine treatment (MGS X E/L in boys; Cognitive, p=.027, F=5 (1,335)). Specifically, robust deleterious effects of magnesium sulfate were seen specifically in boys who *also* went on to receive *early* MX treatment (Cognitive, p=.006, F= 7.6 (1,207); Language, p=.018, F=5.7 (1,206); Motor, *p=.078*, F=3.2 (1,81), Figure 1). Boys that received late *or* no MX showed no overall significant effects of MGS (positive or negative). It is possible that among boys who received *late* MX, perinatal MGS may have provided some anti-inflammatory benefits “cancelling” concurrent deleterious effects -- as evidenced by a loss of Inflammatory Index X Outcome correlations in both girls and boys treated with MGS (Tables 1 and 3). However, negative impacts of MGS were seen in *both* boys with and without a prenatal inflammatory history (Inflam+ boys, MGS effects on Language, *p=.082*, F=3.1 (1,120) (Cog and Motor n.s.), with MGS+ boys doing worse; Inflam- boys, MGS effects on Language, *p=.069*, F=3.3 (1,267), again with MGS+ boys doing worse). These findings suggest the key neurodevelopmental mechanism of MGS action *does not relate to inflammation* -- either positively or negatively.

Moreover, an anti-inflammatory mechanism would not explain a lack of MGS effects in boys with no MX treatment. In further contrast to girls, the deleterious effects of MGS were more robust in *higher* birthweight boys (among boys with birthweight >799 g, MGS effects on Cognitive, p=.023, F=5.2 (1,268), Language, p=.048, F=4 (1,269), with MGS+ boys doing worse; for boys with birthweight <800 g, all values n.s., Figure 1). The reason(s) why MGS may be *particularly* deleterious to boys otherwise predicted to have better outcomes (higher birthweight, early MX) is unclear but does not appear to involve inflammatory mechanisms and stands in contrast to positive MGS effects in girls (particularly those with low birthweight; Figure 1).

To further explore risk/outcome relationships, we also performed Pearson cross-correlations between continuous variables, including birthweight and inflammatory history (using a scale of 0–5), given evidence that these variables significantly impacted mean outcomes when considered categorically. Results showed: (a) Birthweight serves as a highly potent predictor of outcome when considered across Sex, regardless of drug treatments (MX early/late; MGS +/−; Table 1); (b) Inflammatory status is also a robust predictor of outcomes, but only among infants treated with MX later than 48 hrs after birth and/or *not* treated with MGS (Table 1); (c) Both boys and girls showed strong correlations between birthweight and outcomes regardless of MX timing (Table 2), but again, inflammatory status was a predictor only with late MX treatment (particularly in girls; Table 2); (d) Interestingly, birthweight effects on outcomes were reduced in girls exposed to perinatal MGS treatment, consistent with robust MGS benefits specific to low birthweight girls (see above), and weaker birthweight/outcome correlations in MGS+ compared to MGS- girls (Table 3). Inflammatory status also (as in Table 1) was a significant outcome predictor in the absence of MGS treatment for both boys and girls (Table 3).

## Discussion

In summary, we identify several risk factors for *poor* outcomes: low GA/birthweight (with low birthweight being a more potent predictor than low GA); being male; and (in the absence of anti-inflammatory treatment), inflammatory status (defined here as the presence of pre-eclampsia, diabetes, chorioamnionitis, IVH, and/or NEC on a scale of 0–5). We identify several beneficial variables leading to *better* outcomes, including early (<48 hr post birth) MX treatment, particularly for infants with inflammatory history. We also found that magnesium sulfate (MGS) showed some anti-inflammatory benefits as indicated by a loss of inflammation/outcome correlations among infants treated with magnesium sulfate. Moreover, in low birthweight girls, magnesium sulfate led to robust *improvement* in outcomes (with non-significant benefits in higher birthweight girls). However, we also found concomitant *deleterious* effects of magnesium sulfate in boys who went on to receive MX within 48 hrs of birth as well as higher-birthweight boys with late or no caffeine. These paradoxical findings suggest that MGS may impact neurodevelopment via *multiple cellular mechanisms*, with some being beneficial (particularly in girls), but others being deleterious (particularly in boys with early MX treatment and/or higher birthweight). We attempt to reconcile these findings below.

### BIRTHWEIGHT EFFECTS

We were surprised to find that birthweight was a stronger predictor of outcomes than GA. However, this finding likely reflects more complex etiologies of low birthweight, including intra-uterine growth restriction and other factors that deleteriously impact long term outcomes beyond systemic immaturity^28^. We also saw that birthweight was a significant predictor of motor outcomes in girls but not boys. Finally, we were intrigued by evidence that magnesium sulfate could obviate deleterious effects of low birthweight in girls but not boys – a benefit that was *not* seen for methylxanthines.

### INFLAMMATORY EFFECTS

It is well established that inflammatory pre- or postnatal events can exert deleterious effects on neurodevelopment^29, 30, 31^. Here we report strong evidence that early MX treatment can mitigate these deleterious inflammatory effects in both sexes, with MX treatment largely eliminating inflammatory relationships to outcomes in both sexes (Tables 1,2). Similar loss of inflammatory/outcome correlations were seen with MGS treatment (Tables 1,3), although ANCOVA results clearly revealed that MGS+ outcomes were improved *only* for females. Moreover, both beneficial MGS effects in girls and deleterious effects of MGS in boys were seen *irrespective of inflammatory status*. This suggests while MGS may have some anti-inflammatory properties, critical effects on neurodevelopmental outcomes likely involve parallel *non*-inflammatory mechanisms. Moreover, the fact that MGS+ interacts negatively with concomitant (early) MX in boys further suggests that MGS may be acting on competing or overlapping cellular pathways to MX, at least in boys. This in turn could reflect sex differences in critical cell death pathways, with sex differences in therapeutic benefit or harm depending on sex-specific targets of action^32 – 35^.

### SEX DIFFERENCES

The overall benefit among preterms of being female is well established^36^. The fact that magnesium sulfate appears to show *specific* benefit for girls and not boys has also been reported previously^37^. This sex difference could reflect a variety of factors, including differential developmental trajectories of glutamatergic systems as a function of sex^38, 39^, and/or sex differences in activation of cell death pathways which could in turn be differentially impacted by MGS in males and females. Developmental trajectory differences in NMDA systems could also explain why magnesium sulfate benefits were more robust for low birthweight girls, who are likely developmentally more immature. MX benefits, conversely, appear to occur regardless of sex, similar to reports for hypothermia (“cooling“) in term infants with hypoxic-ischemic encephalopathy (HIE) where benefits are also seen regardless of sex^40^.

### BENEFITS OF METHYXANTHINES/CAFFEINE

Benefits of methylxanthine (MX) treatment for the preterm infant outcomes are now well established. Our results confirm MX as a potent modulator of the deleterious effects of inflammation in both sexes, although MX does not appear to obviate the negative effects of low birthweight in either sex. The fact that MX does *not* appear to interact with sex favors this drug class (including caffeine) as a more viable preterm neuroprotective candidate than MGS -- at least in cases where maternal pre-eclampsia is not a specific risk factor. With regards to MX mechanisms of action, effects are thought to occur via adenosine-receptor (AR) antagonism, as well as concurrent action as a phosphodiesterase inhibitor and an active intracellular calcium mobilizer^41^. MX-class drugs are nonselective adenosine antagonists that can bind to four neuronal adenosine receptor subtypes (A1, A2A, A2B, and A3). This is important because adenosine levels rise in response to cerebral hypoxia, leading to increased cerebral inflammation and heightened immune response. Adenosine receptors in turn serve as important modulators of the resulting cell-death cascade^42^. Neuroprotective MX effects are thought to be mediated by antagonism at either A1 and/or A2A receptor sites, where they are thought to prevent excess calcium influx and subsequent release of protein-bound intracellular calcium — ultimately reducing cell death (43). The A2AR is also present in microglia, and antagonism here reduces microglial activation, as well as inflammation -- again mitigating cell death and tissue loss^44^. Interestingly, animal studies have shown that caffeine decreases microglial activation after HI injury, *but only in male rats*^45^. Nonetheless, both male and female preterm rat pups show comparable neuroprotection. This implies that MX (including caffeine) exerts protective effects via *different* adenosine receptor sites and mechanisms in females and males — a sex difference that could further explain the selective deleterious effects of MGS treatment combined with early MX only in males, but not females.

### MIXED EFFECTS OF MAGNESIUM SULFATE

Although magnesium sulfate treatment appeared to counter inflammatory effects on outcomes in both sexes (as evidenced by loss of correlations between inflammatory status and outcomes in treated groups), nonetheless positive MGS effects on outcomes were only seen in girls. This suggests some other deleterious mechanism of action in boys (and particularly those with early MX, or late/no MX but higher birthweight). Moreover, the fact that beneficial MGS effects were seen in female preterms regardless of positive or negative inflammatory status (and negative effects in boys regardless of inflammatory status) indicates that the primary effects of MGS on neurodevelopmental outcomes are *not* mediated by anti-inflammatory action.

Instead, MGS may be directly targeting excitotoxic (e.g., glutamatergic) pathways in the fetal/neonatal brain, including the NMDA receptor and ion channel which are rapidly changing in the fetus during the last trimester of pregnancy^46–50^. Moreover, given observed sex differences in glutamatergic maturational trajectories, these effects could include preferential benefit to females. Robust sex differences in MGS therapeutic benefit/harm could also reflect well-established sex differences in the activation of caspase-dependent versus independent cascades of excitotoxicity and neuronal cell death^51^. Specifically, since females favor caspase-dependent cell death pathways following insult, these pathways may be selectively blocked or attenuated by MGS treatment. Conversely, males show elevated activation of caspase-independent cell death cascades, where MGS treatment (and associated NMDA-blockade) could selectively enhance *deleterious* excitotoxicity. Indeed, male neonates show more excitatory NMDA receptors overall, making them more vulnerable to hypoxia and other events that activate the NMDA receptor^52^. These sexually dimorphic trajectory differences in NMDA systems could further explain why MGS is particularly *beneficial* to low-birth-weight (less developed) females, and particularly *deleterious* to high-birthweight (more developed) males.

There is also evidence from rodent studies that in the absence of injury, MGS may in general cause enhanced cell death and reduced plasticity^53, 54^. This may mean that in cases of low risk of infant injury, MGS may cause more harm than benefit — especially for males. This, in turn, could relate to significantly elevated levels of circulating androgen in the third trimester male fetus, since androgens have been associated with enhanced excitotoxicity^55^. These findings could also relate to stronger MGS impacts in higher birthweight boys.

Finally, with regards to MX x MGS interactions in boys, evidence shows that magnesium in conjunction with caffeine leads to an influx of calcium into the brain cell which is generally toxic^56^. In addition, magnesium appears to interfere with adenosine receptors (AR1 and AR2), which could preclude the normal interaction of adenosine blockers such as caffeine^57^. Either or both could explain why negative MGS effects in boys are particularly exacerbated among those receiving early methylxanthines, and suggest a complex pharmacologic MX x MGS interaction in boys which may be minimized by waiting until 48 hours after birth to administer caffeine to male preterms treated with MGS -- allowing for clearance, and minimizing drug interactions.

### LIMITATIONS

We acknowledge several limitations in the current study. First, many of the infants in the current dataset received multiple drug treatments (as dictated by medical needs). Although inclusion of these infants allowed us to identify heretofore unreported MX x MGS interactions, it also confounds reporting of individual drug effects. Second, infants likely received additional drugs that we did not assess (e.g., antenatal steroids, thyroxine, etc.) and these should be included in future studies. Finally, our findings derive from a single-site dataset and lack information on familial SES, as well as other key variables pertinent to infant outcomes. Future analyses should endeavor to include a more heterogenous preterm sample and should include SES information as a covariate.

## Conclusions

Our primary findings are as follows. (1) Methyxanthines (MX, such as caffeine) primarily benefit infants with some inflammatory history, consistent with anti-inflammatory MX mechanisms. These benefits occur for both boys and girls, with no apparent deleterious effects *except* in males with prior MGS exposure. (2) Magnesium sulfate (MGS) appears to exert neurodevelopmental effects *independent* of inflammation, with benefits specific to girls (and more specifically low-birthweight girls, although there are no deleterious effects in higher birthweight girls). MGS effects, conversely, are largely ineffective or deleterious in boys -- especially those with early MX treatment, or late/no MX but higher birthweight. Collective findings add more nuance and important qualifications to the current literature, wherein both caffeine and magnesium sulfate are reported as viable neuroprotective options in preterm deliveries *when sex and birthweight are ignored*^58^. Findings also highlight the critical importance of fetal/neonatal sex in treatment considerations, consistent with evidence of dramatic differences in pharmacokinetics and drug metabolism in adult men and women^59, 60^. It should not be seen as surprising that at-risk newborns have similarly heterogeneous responses to commonly used perinatal medications. This over-looked area of neonatal medicine emphasizes the need for additional research to optimize therapeutic interventions in early deliveries, particularly where time allows for considered deliberation.

In sum, findings support hastening the provision of MX treatment in almost all preterm deliveries, especially those with inflammatory clinical signs, but with a clear exception for higher-birthweight male neonates previously exposed to magnesium sulfate. Findings also highlight the benefits of magnesium sulfate treatment for mothers expected to deliver a low-birthweight (<800 g) female, while emphasizing contraindications for magnesium sulfate use in delivery of males where pre-eclampsia is not a consideration (particularly higher birthweight males) along with a >48 hour delay in MX/caffeine administration for male neonates when magnesium sulfate has been used.

## Figures and Tables

**Table 1.** Pearson bivariate correlations between birthweight, inflammatory index, and outcomes. Subgroups (combined sexes): Late (> 48 hrs after birth) methyxanthines (MX); Early (< 48 hrs) MX; no magnesium sulfate (MGS− including early, late and no MX); and MGS+ (including early, late and no MX).

	*Late MX*		*Early MX*		*MGS−*		*MGS+*	
	BW	INFL	BW	INFL	BW	INFL	BW	INFL
Cognitive	.009*** (260)	.033* (260)	.008** (428)	.6 (ns) (428)	.001**** (301)	*.06*# (301)	.003*** (475)	.85 (ns) (475)
Language	.018* (261)	.02* (261)	.25 (ns) (431)	.8 (ns) (431)	.14 (ns) (302)	.03* (302)	.037* (478)	.8 (ns) (478)
Motor	.4 (ns) (112)	.017* (112)	.001**** (170)	.5 (ns) (170)	.012* (90)	.005*** (90)	.008** (196)	.85 (ns) (196)

Green=sig/positive; Pink=sig/negative; BW = birthweight; INFL = inflammation index (0–5); MX = methyxanthine; MGS= magnesium sulfate; cell value includes Pearson R p-level and (n).p<.1#, .05*, .01**, .005***, .001****

**Table 2.** Birthweight and inflammatory index correlations to outcomes in boys and girls with late (> 48 hrs after birth) versus early (< 48 hrs after birth) methyxanthine (MX).

	*Late MX, boys*		*Early MX, boys*		*Late MX, girls*		*Early MX, girls*	
	M BW	M INFL	M BW	M INFL	F BW	F INFL	F BW	F INFL
Cognitive	.045* (135)	.183 (ns) (135)	.035* (210)	.9 (ns) (210)	.048 * (125)	*.072*# (125)	.03* (218)	.65 (ns) (218)
Language	.45 (ns) (135)	.24 (ns) (135)	.37(ns) (211)	.6 (ns) (211)	.006** (126)	.036* (126)	.42 (ns) (220)	.73 (ns) (220)
Motor	.54 (ns) (61)	*.1*# (61)	.02* (84)	.4 (ns) (84)	.037* (51)	*.084*# (51)	.004*** (86)	.87 (ns) (86)

Green=sig/positive; Pink=sig/negative; BW = birthweight; INFL = inflammation index (0–5); MX = methyxanthine; MGS= magnesium sulfate; cell value includes Pearson R p-level and (n).p<.1#, .05*, .01**, .005***, .001****

**Table 3.** Birthweight and inflammatory index correlations to outcomes in boys and girls with or without magnesium sulfate (MGS) treatment.

	*MGS− boys*		*MGS+ boys*		*MGS− girls*		*MGS+ girls*	
	M BW	M INFL	M BW	M INFL	F BW	F INFL	F BW	F INFL
Cognitive	*.053*# (158)	.3 (ns) (158)	.014* (234)	.77 (ns) (234)	.004*** (143)	*.095*# (143)	.025* (241)	.64 (ns) (241)
Language	.75 (ns) (158)	.13 (ns) (158)	*.07*# (235)	.76 (ns) (235)	.03* (144)	*.066*# (144)	.15 (ns) (243)	.87 (ns) (243)
Motor	.7 (ns) (52)	.009** (52)	.18 (ns) (95)	.63 (ns) (95)	.008** (38)	.12 (ns) (38)	.012* (101)	.79 (ns) (101)

Green=sig/positive; Pink=sig/negative; BW = birthweight; INFL = inflammation index (0–5); MX = methyxanthine; MGS= magnesium sulfate; cell value includes Pearson R p-level and (n).p<.1#, .05*, .01**, .005***, .001****

## Abbreviations

AR: adenosine receptor
BW: birthweight
CAT/CLAMS: Cognitive Adaptive Test/Clinical Linguistic Auditory Milestone Scale
CP: cerebral palsy
CCMC: Connecticut Children’s Medical Center
E/L: early/late
GA: gestational age
HIE: hypoxic-ischemic encephalopathy
IVH-PVH: intra-ventricular hemorrhage/periventricular hemorrhage
MGS: magnesium sulfate
MX: methyxanthine (caffeine, theophylline)
NEC: necrotizing enterocolitis
NICU: neonatal intensive care unit
NMDA: N-methyl-D-aspartate
UCHC: University of Connecticut Health Center
